# What is the effect of alarmist media and radiofrequency electromagnetic field (RF‐EMF) exposure on salivary cortisol and non‐specific symptoms?

**DOI:** 10.1111/aphw.70044

**Published:** 2025-05-28

**Authors:** Adam Verrender, Nikkeah K. Wallace, Sarah P. Loughran, Chloe Wallace, James Beange, Rodney J. Croft

**Affiliations:** ^1^ Australian Centre for Electromagnetic Bioeffects Research Wollongong Australia; ^2^ School of Psychology, Faculty of Arts, Social Sciences, and Humanities University of Wollongong Wollongong Australia; ^3^ Australian Radiation Protection and Nuclear Safety Agency Yallambie Australia

**Keywords:** cortisol, electromagnetic hypersensitivity, idiopathic environmental intolerance, nocebo effect, radiofrequency electromagnetic fields, symptoms

## Abstract

While there is consistent evidence that the symptoms reported by people who experience Idiopathic Environmental Intolerance attributed to Electromagnetic Fields (IEI‐EMF) are closely associated with a nocebo effect, and that alarmist media reports may contribute to this nocebo effect, some methodological criticisms remain to be resolved. This study aimed to replicate previous findings and determine whether viewing an alarmist media report and being openly exposed to radiofrequency electromagnetic fields (RF‐EMF) could induce a salivary cortisol response. A total of 144 participants were randomly assigned to watch either an alarmist or control video before completing an open‐label provocation trial where they were either exposed or not exposed to RF‐EMF. Personality factors, RF‐EMF risk perception (pre‐ and post‐video), symptoms and salivary cortisol were assessed. Consistent with previous studies, participants who were aware that they were being exposed had increased symptoms compared to participants who were aware they were not being exposed. However, the current study failed to replicate an effect of viewing an alarmist media report and being openly exposed to RF‐EMF on symptoms and failed to identify an effect on salivary cortisol. This suggests that awareness and belief of exposure play a more important role in symptom perception than underlying physiological processes.

## INTRODUCTION

Over the past few decades, the use of wireless technologies has rapidly increased with the continuing development of mobile phone, laptop, tablet, and smart devices. For instance, in 2023, the United Nations estimated that there were 8.9 billion active mobile phone subscriptions, a figure which exceeds the world population (International Telecommunications Union, 2023). However, while advancements in wireless technologies have transformed society, many people remain concerned that the radiofrequency electromagnetic fields (RF‐EMF) emitted by these devices can cause adverse health effects (Wiedemann et al., 2017). Among these are approximately 3–5% of the population who claim to experience a wide range of non‐specific symptoms which they believe are caused by exposure to RF‐EMF (Baliatsas et al., 2012; Huang et al., 2018; Schmiedchen et al., 2019). These individuals suffer from a condition commonly referred to as Electromagnetic Hypersensitivity (EHS). As there is no evidence that exposure to RF‐EMF below international safety guidelines (e.g., ICNIRP, 2020) can adversely affect health (SCENIHR, 2015), and no clear bioelectromagnetic process has been shown to contribute to the development of these symptoms, the World Health Organisation has recommended the term Idiopathic Environmental Intolerance attributed to Electromagnetic Fields (IEI‐EMF) be used to describe this condition (Bosch‐Capblanch et al., 2024).

The majority of well‐controlled, double‐blind provocation studies have found that sham RF‐EMF exposure is sufficient to trigger symptoms reported by IEI‐EMF sufferers (Hillert et al., 2008; Oftedal et al., 2007; Rubin et al., 2010; Verrender, Loughran, Anderson, et al., 2018). In addition, studies that have utilized both open‐label and double‐blind trials have found that symptom severity increases when participants are *aware* that they are being exposed to active RF‐EMF (compared to a control condition) in open‐label trials, but in subsequent double‐blind trials where both participants and researchers are *unaware* of the exposure condition, there is no difference in symptom severity between active and sham conditions (Eltiti et al., 2007; van Moorselaar et al., 2017; Verrender, Loughran, Anderson, et al., 2018). These results suggest that the condition is closely associated with a nocebo effect, as awareness of the exposure condition and a belief of being exposed have been shown to play a crucial role in the presentation of symptoms (Bosch‐Capblanch et al., 2024; Rubin et al., 2010; Schmiedchen et al., 2019).

Interestingly, recent studies have suggested that alarmist media reports emphasizing adverse effects of RF‐EMF exposure may contribute to this nocebo effect, and these alarmist media reports have been found to increase symptoms in healthy participants (Bräscher et al., 2017; Verrender, Loughran, Dalecki, et al., 2018; Witthöft & Rubin, 2013). For example, in a previous study, we tested whether perceived EMF exposure could elicit symptoms in healthy participants and whether viewing an alarmist media report could exacerbate a nocebo effect (Verrender, Loughran, Dalecki, et al., 2018). The results showed that the belief of being exposed, rather than the RF‐EMF exposure itself, was sufficient to trigger symptoms in healthy participants. Furthermore, participants who viewed the alarmist video had a significant increase in symptoms relative to the control video group. Again, these results demonstrate the crucial role of awareness/belief of exposure in the presentation of symptoms and suggest that alarmist media reports that emphasize adverse effects of RF‐EMF exposure may contribute to a nocebo effect.

Although the results of our previous study align with the broader IEI‐EMF research (Bosch‐Capblanch et al., 2024; Bräscher et al., 2017; Rubin et al., 2010; Witthöft & Rubin, 2013), the subjective measures used in IEI‐EMF provocation studies have been criticized for producing potentially unreliable data that may be influenced by bias (Bräscher et al., 2020; Leszczynski, 2021). While there is little evidence to suggest that objective measures can provide a more accurate or clinically meaningful picture than subjective symptom measures for those who experience IEI‐EMF, especially given that adverse non‐specific symptoms are the most common complaints reported by IEI‐EMF sufferers, it may be useful to determine whether an objective measure can provide complementary information that could further our understanding of IEI‐EMF (Adewusi et al., 2021).

Studies investigating the possibility of physiological correlates of nocebo effects mostly stem from the investigation of pain processing in healthy participants. For instance, Benedetti et al. (2006, 2007) found that negative verbal suggestions following the administration of inert substances can induce anticipatory anxiety about the impending pain increase, which, in turn, activates two different and independent biochemical pathways. One pathway involves the activation of cholecystokinin (CCK), a neuropeptide that has been found to play a crucial role in a number of psychological and physiological functions (Hebb et al., 2005), including as a neuromodulator in the experience of pain (Benedetti et al., 2007). The other pathway involves the activation of the hypothalamic–pituitary–adrenal axis (HPA), a pathway implicated in the release of cortisol and the experience of stress and anxiety (Benedetti et al., 2006, 2007). While stress is known to precipitate, exacerbate, and perpetuate physical symptoms (McEwen, 1998; Nater et al., 2013), the role of stress in the presentation of symptoms attributed to RF‐EMF has not been determined. The HPA axis is a system that is particularly responsive to psychosocial stress (Nater et al., 2013), and its activation can be readily measured via salivary cortisol. If HPA activity is affected by explicit suggestions of harm via alarmist media reports, or the experience of an EMF exposure situation that is perceived as threatening, this may explain the presentation and exacerbation of symptoms reported by people who experience IEI‐EMF and may provide a more objective measure of the nocebo effect associated with RF‐EMF exposure. Conversely, it is also conceivable that RF‐EMF could affect cortisol directly via thermoregulatory processes. For instance, Cameron et al. (2010) found that the concentration of bioavailable free cortisol in‐vitro is influenced by increases in temperature. Although this effect has not been demonstrated in humans, it is possible that increases in body temperature can affect cortisol concentration. While the ICNIRP (2020) exposure guidelines have been designed to protect against adverse health effects caused by RF‐EMF‐derived heating, it has been recently demonstrated that RF‐EMF below the ICNIRP guidelines can still engage human thermoregulatory processes (Loughran et al., 2019). This means that it is possible that a small increase in body temperature derived from RF‐EMF may affect cortisol concentration, however, this has yet to be tested. If cortisol concentration is increased by small RF‐EMF‐induced temperature increases, this may also explain the presentation and exacerbation of symptoms reported by people who experience IEI‐EMF. As stress responses and elevated cortisol can affect several physiological systems in humans (Yaribeygi et al., 2017) and may result in the perception of symptoms, it is important to determine whether this physiological mechanism is more generally involved in the presentation of symptoms attributed to RF‐EMF exposure. Although previous studies have shown clear evidence that alarmist media reports promoting claims that RF‐EMF can cause adverse health effects can contribute to the experience of a nocebo effect, whether these alarmist reports continue to be relevant to a population that is increasingly using wireless technologies remains unclear. For instance, a recent study found that viewing a film promoting claims that EMF exposure causes adverse health effects did not enhance participants self‐reported ratings of intensity or aversiveness of an electrodermal stimulus after being exposed to a Sham Wi‐Fi condition (i.e. a nocebo effect) compared to viewing a control video (Bräscher et al., 2020). Instead, the information given by researchers and the experimental context were suggested to be more important for inducing a nocebo effect, as the film may have aged and been less relevant to the participants, given that it was first broadcast in 2010 (Bräscher et al., 2020). This corresponds with recent analysis suggesting that the decline in the prevalence of IEI‐EMF in some countries may be partially explained by the decrease in the number of media reports focusing on the condition, and the efforts of scientists, health workers, industry, and government agencies to communicate the scientific evidence more clearly to alleviate the public's fear of EMF exposure (Huang et al., 2018).

It is also possible that both situational factors (such as viewing a particular media report) and dispositional factors (such as personality traits) interact to influence a nocebo effect. If certain personality traits contribute to an individual being more susceptible to experiencing nocebo effects, a useful clinical application would be the ability to identify these traits and develop tailored, effective interventions. However, the personality traits that may be involved in moderating nocebo effects remain to be clarified. For instance, there has been mixed evidence about the moderating role of state and trait anxiety in the propensity for individuals to experience nocebo effects (Kern et al., 2020; Rooney et al., 2023; Webster et al., 2016). In our previous study (Verrender, Loughran, Dalecki, et al., 2018), pre‐existing levels of state anxiety did not influence whether participants experienced a nocebo effect. This finding was in contrast to Witthöft and Rubin (2013), who reported that participants with higher pre‐existing levels of state anxiety experienced increased symptoms. Similarly, neuroticism, which is broadly conceptualized as having a tendency to exhibit anxiety and be vulnerable to stress (Costa & McCrae, 1985), has been found to both increase and decrease psychogenic symptom reporting (Davis et al., 1995; Mazzoni et al., 2010). Interestingly, Feldman et al. (1999) found that people high in neuroticism were more likely to interpret physiological cues as threatening and distressing than those with lower neuroticism. The interpretation of cues as threatening may impact the experience of a nocebo effect, as people with high neuroticism may have learned that bodily sensations felt after the administration of a treatment or intervention (but not caused by them) should be perceived as overly negative. This, however, is speculative. Clearly, further investigation is needed to clarify whether state anxiety, trait anxiety, or neuroticism are associated with a nocebo effect.

To address these issues, the current study aimed to replicate the results of our previous study (Verrender, Loughran, Dalecki, et al., 2018) and extended this to determine whether being openly exposed to RF‐EMF after viewing an alarmist video can induce an associated cortisol response in healthy participants. It tested the hypothesis that 1) viewing an alarmist video and being openly exposed to RF‐EMF increases symptoms in healthy participants, and 2) viewing an alarmist video and being openly exposed to RF‐EMF increases salivary cortisol in healthy participants. The measurement of salivary cortisol levels may overcome the claimed limitations of IEI‐EMF research by offering a more objective index of physiological stress and the experience of a nocebo effect, rather than relying on self‐reported symptom severity. The present study also sought to explore whether individual personality factors are associated with the experience of a nocebo effect by testing whether state anxiety, trait anxiety, and/or neuroticism were related to increases in symptom score.

## MATERIALS AND METHOD

### Participants

One hundred and fifty‐two participants (65% Female, 35% Male) aged 18–41 (*M =* 21.32, *SD* = 3.79) were recruited through online advertisements, advertisements placed around the University of Wollongong campus, the School of Psychology Research Participation Scheme, and word of mouth. An a priori power calculation conducted in G*Power 3.1 (Faul et al., 2007) determined that a minimum sample size of 128 was required to provide sufficient power (0.80) to detect effect sizes (Cohen's f) of greater than 0.25. This provides enough power to detect a main effect of belief of RF‐EMF exposure on symptoms and an interaction between video group and RF‐EMF exposure on symptoms consistent with those found in the literature (Verrender, Loughran, Dalecki, et al., 2018). For the main effect of the video on cortisol response, the minimum sample provides the power (0.80) to detect effect sizes of greater than 0.25, based on the effect found by similar paradigms using video content to elicit a cortisol response (Nejtek, 2002), with the exception that the current study also involved a provocation trial, rather than a passive video‐watching task only.

To be included in the study, participants were required to be between 18 and 55 years of age, be sufficiently fluent in English, and report being of good health. Participants were excluded from the study if they reported that they were receiving treatment for or suffered from any acute or chronic illnesses, or if they reported taking any pharmaceutical or illicit substances. Participants who were deemed suitable subsequently attended the University of Wollongong campus for one mutually convenient time for testing. Informed, written consent was acquired from all participants. The study was approved by the University of Wollongong Human Research Ethics Committee (HE: 2022/146). Participants were compensated with a monetary gift card (general participants) or credit points (Psychology students).

### Design

**FIGURE 1:**
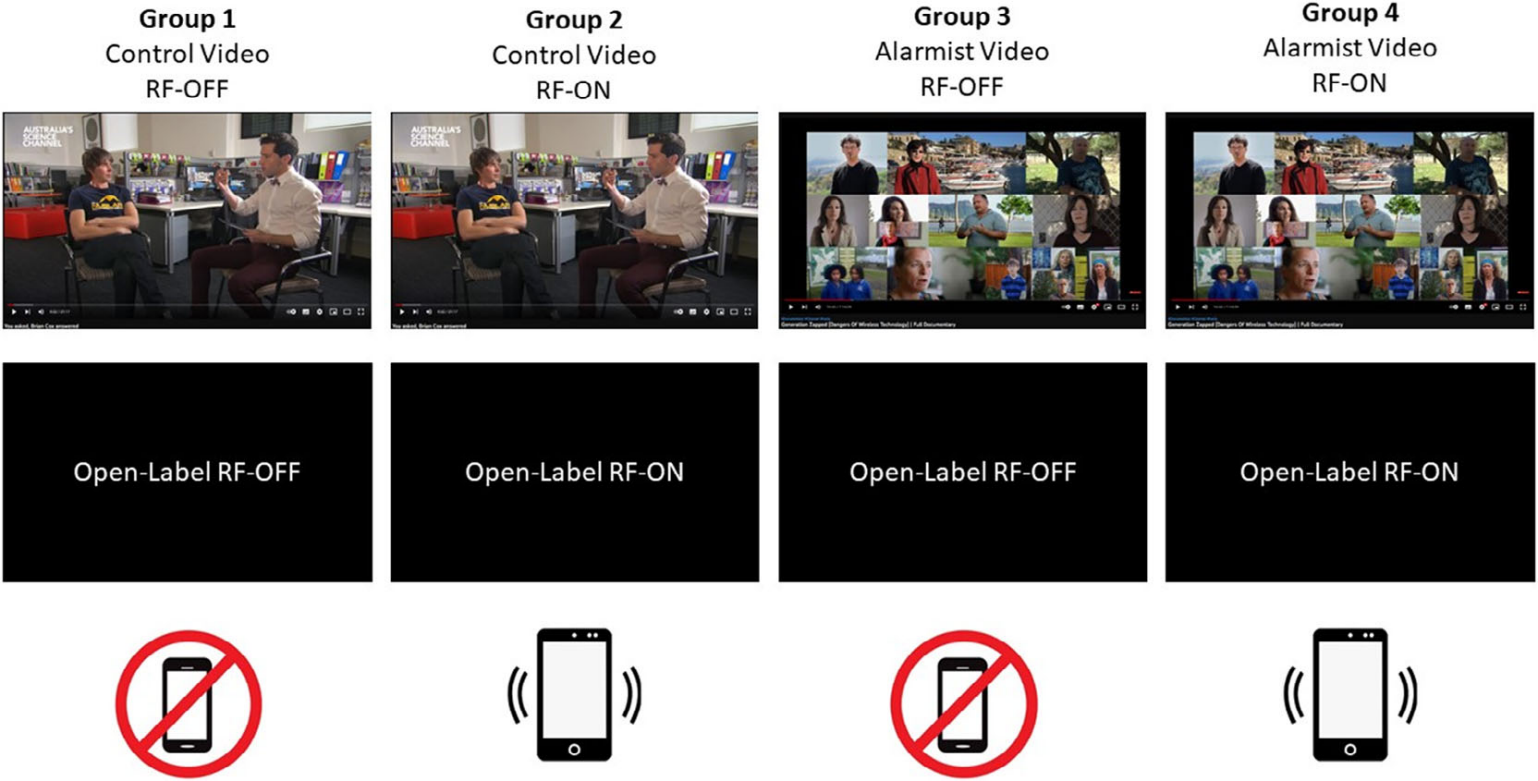
Participants were randomly allocated to one of four experimental groups, where they were assigned to watch either a control (group 1 and 2) or alarmist video (group 3 and 4) and were either not exposed (RF‐OFF; group 1 and 3) or exposed (RF‐ON; group 2 and 4) to RF‐EMF.

A randomized, counterbalanced, between‐groups design was utilized in this study. Prior to attendance, participants were randomly allocated into one of four experimental groups by a computerized process, where group allocation was pseudo‐randomly assigned to blocks of four participants using the rand() function in Microsoft Excel. To control for any possible effect of group allocation order on gender, blocks of males and females received the same group allocation (ie, Male Participants 1–4 received the same group allocation as Female Participants 1–4, etc.). Participants were assigned to watch either an alarmist or a control video and were either exposed (RF‐ON) or not exposed (RF‐OFF) in an open‐label provocation trial, as illustrated in Figure 1.

Participants assigned to the control video groups (Group 1 and Group 2) viewed a 15‐minute segment of a science Q and A documentary titled “You asked, Brian Cox answered.” This video contained an interview with two astrophysicists answering students' questions about the universe and contained no RF‐EMF health‐related content (The Royal Institute of Australia, 2016). Participants assigned to the alarmist video groups (Group 3 and Group 4) viewed the first 15‐minute segment of a video titled “Generation Zapped: Dangers of Wireless Technology.” This video contained interviews with scientists, IEI‐EMF sufferers, and members of the community who expressed strong, explicit concerns that RF‐EMF exposure caused adverse health effects, including claims that exposure to RF‐EMF causes cancer and IEI‐EMF (El Gemayel, 2017).

For the provocation trials, participants belief of exposure status and symptoms were tested in a single, non‐blinded open‐label trial (RF‐OFF or RF‐ON) where both the participant and the researcher were aware of the exposure condition, which was demonstrated to the participant using a Nardalert S3 broadband monitor (Narda Safety Test Solutions, Hauppauge, NY). In the RF‐ON condition, the broadband monitor would beep and flash, and show colored percentage bars indicating low and high frequency exposure. In the RF‐OFF condition, the broadband monitor was silent and did not indicate any low or high frequency exposure.

### Radiofrequency exposure

A 920 MHz GSM‐like signal (as emitted by a mobile phone in an active mode while transmitting voice) was generated using an sXh920 planar exposure system (IT'IS Foundation, Zurich, Switzerland). Two RF antennas placed on wooden pillars were positioned 42 mm vertically above the ear canal at a distance of 115 mm from the head laterally (Loughran et al., 2019; Verrender et al., 2016). The RF exposure has been fully characterized and was calibrated to provide a peak‐spatial SAR averaged over 10 g of 0 W/kg and 2 W/kg for the RF‐OFF and RF‐ON conditions, respectively (for full dosimetric data see Murbach et al. (2012)). The sXh920 system enables precise exposure levels and has a failsafe mechanism to ensure that it cannot exceed the relevant RF general public exposure limits (ARPANSA RPS‐S1). The exposure was provided within a Faraday cage with 80 dB attenuation, which minimized any environmental EMF exposure from nearby EMF‐emitting devices such as mobile phones, Wi‐Fi, and mobile phone base stations.

### Salivary cortisol measures

**FIGURE 2:**
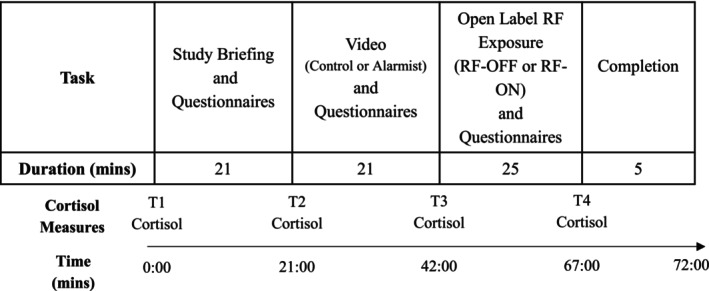
Trial procedure. Participants first provided a baseline saliva sample (TI cortisol) before completing the study briefing and the first battery of questionnaires (STAI‐Y1, STAY‐Y2, NEO‐FFI, RPQ). After providing a second saliva sample (T2 cortisol) participants watched their assigned video (control or alarmist) and completed a second battery of questionnaires (STAI‐Y1, RPQ). Following this, participants provided a third saliva sample (T3 cortisol) and completed the provocation trial (RF‐OFF or RF‐ON), where symptoms were assessed using the SESS at three intervals. At the conclusion of the provocation trial, participants provided a fourth saliva sample (T4 cortisol) and completed the final battery of questionnaires (STAI‐Y1, RPQ).

Whole saliva samples were collected using the passive drool method (SalivaBio 2 mL cryovials and Saliva Collection Aid; Salimetrics, USA). Saliva samples were collected at four time points 21 minutes from the onset of each interval (except baseline); see Figure 2. Saliva samples were immediately stored in a freezer at −20°C and were then transferred to a − 80°C freezer. Whole saliva collected by passive drool is the gold standard when collecting oral fluid for biological testing. It avoids localized secretions of specific salivary glands, which provides a more consistent specimen, and as it is free from being compromised by absorbing‐materials used with other collection methods, whole saliva can be assayed for all analytes of interest. The samples were sent to Stratech Scientific salivary laboratory (Stratech Scientific APAC Pty Ltd, Sydney, Australia) for assay. On the day of assay, the samples were thawed to room temperature, centrifuged at 1500 x *g* for 15 minutes to collect clear saliva, and analyzed in duplicate using a commercially available ELISA assay (Salimetrics, USA) according to the manufacturer's instructions. The primary dependent variable of cortisol response in the provocation trial was calculated as a difference score (in μg/dL) between the baseline and exposure intervals (Time 4 Cortisol minus Time 1 Cortisol; see Figure 2); a difference score was used to minimize the influence of baseline variability.

### Questionnaires

#### Symptoms and exposure status scale (SESS)

During the provocation trials, participants were asked to indicate whether they believed the exposure was on or off, and to indicate the extent to which they were experiencing any symptoms via pen and paper 100 mm visual analogue scales. To assess belief of exposure, participants were asked “how sure are you of the current exposure status right now?” anchored with the terms ‘Definitely OFF’ and ‘Definitely ON’. To assess symptom experience, a modified state version of the 34‐item Checklist for Symptoms in Daily Life (Wientjes & Grossman, 1994; Witthöft & Rubin, 2013) was used. Participants were asked “how strong/unpleasant are the following symptoms right now?” anchored with the terms ‘Barely Detectable’ and ‘Maximum Severity’. These response categories differed from the original questionnaire (Wientjes & Grossman, 1994) and were used in line with our previous studies (Verrender, Loughran, Anderson, et al., 2018; Verrender, Loughran, Dalecki, et al., 2018). The symptom responses (scored out of 100) of each of the 34 items were added to calculate a total symptom score for each of the baseline and exposure intervals in each trial. The primary dependent variable of symptom score in the provocation trial was calculated as the difference between the baseline and exposure questionnaires (exposure interval minus preceding baseline interval); a difference score was used to minimize the influence of baseline variability.

#### Risk perception questionnaire (RPQ)

A risk perception questionnaire comprising 4 questions was used to assess EMF risk perception (Verrender, Loughran, Anderson, et al., 2018; Verrender, Loughran, Dalecki, et al., 2018). Question 1 assessed concerns about electromagnetic fields in general, and question 2 assessed concerns about electromagnetic fields in relation to mobile phones and Wi‐Fi. In question 1, participants were asked “How concerned are you about the potential health risks of electromagnetic fields in general?” rated on a 7‐point Likert scale (1 = not worried at all, 7 = very worried) and in question 2, participants were asked “All in all, how threatened do you feel by electromagnetic radiation emissions from mobile phones and Wi‐Fi?” rated on a 7‐point Likert scale (1 = not threatening at all, 7 = very threatening). To aid participants' assessment of RF‐EMF risk perception in relation to mobile phones and Wi‐Fi, questions 3 and 4 used picture‐guided scenarios that illustrated everyday exposure situations (Freudenstein et al., 2015). In question 3 participants were asked “How dangerous do you think the electromagnetic fields from mobile phones are while you talk on the phone, as illustrated in this picture?” and in question 4, participants were asked “How dangerous do you think the electromagnetic fields are from Wi‐Fi routers in close proximity, as illustrated in this picture?” Questions 3 and 4 were rated on a 7‐point Likert scale (1 = not dangerous at all, 7 = very dangerous). The RPQ score was defined as the mean score from the 4 responses.

#### State and trait anxiety index (STAI)

The 40‐item version of the STAI (Spielberger et al., 1970) was used to assess participant's state and trait anxiety. This comprises two, 20‐item forms, assessing state (STAI‐Y1) and trait (STAI‐Y2) anxiety separately, with items answered on a 4‐point Likert scale (1 = not at all, 4 = very much so).

#### NEO five factor personality index (NEO FFI)

The 60‐item NEO FFI (Costa & McCrae, 1992) was used to assess personality traits: Extraversion, Agreeableness, Conscientiousness, Neuroticism, and Openness to Experience.

### Procedure

A participant information sheet was sent to interested people who responded to the study advertisements and contacted the researchers. This sheet informed participants that a proportion of the population report being sensitive to and/or experiencing a range of symptoms which they attribute to EMF, described some of the symptoms and devices people report being sensitive to, and explained that although the scientific evidence has yet to establish a clear relationship between exposure and symptoms, media reports about the possible adverse health effects of RF‐EMF exposure continue to focus on people who report these symptoms. The information sheet also included statements about the general aims of the study, which outlined that the first part of the study would assess the effect of media communication on risk perception and physiology, and the second part of the study would investigate whether there is a relationship between short term RF‐EMF exposure, symptoms, and physiology in the general population.

Once determined to be eligible via a telephone screening interview, participants were booked in for one mutually convenient testing session starting at either 12 pm, 1 pm, 2 pm, or 3 pm, which lasted approximately 1 hour and 15 minutes. Before arriving at the laboratory, participants were instructed to abstain from consuming caffeine, food, and drinks (including water) for 1 hour, and alcohol for 8 hours prior to the commencement of their session, as well as to abstain from making mobile phone voice calls for 2 hours, and to obtain a full night's sleep (7–9 hours) before the commencement of their session. Participants were reminded of these instructions several times, including in the Participant Information Sheet, in the initial telephone screening, in the confirmation email, and in the reminder email sent 24 hours before participation.

The subsequent procedure for the trial is outlined in Figure 2. Upon arrival at the laboratory, participants provided informed written consent. Participants then provided a baseline saliva sample (Time 1 Cortisol). To obtain a saliva sample, participants were instructed to allow saliva to pool in the mouth and then tilt the head forward gently, wrap their lips over the Saliva Collection Aid, and gently guide saliva through the Saliva Collection Aid into the cryotube. Once it was determined that participants understood the instructions for providing a saliva sample, the researchers left the room to allow the participants privacy. Once the sample was provided, the researchers gave a briefing of the ensuing session. This briefing included telling participants that they would be watching a film, and explicitly telling them whether they would or would not be exposed to RF‐EMF. Following the study briefing, participants completed a battery of questionnaires assessing baseline risk perception (RPQ), state (STAI‐Y1) and trait anxiety (STAI‐Y2), and personality (NEO‐FFI). Once the questionnaires were completed, participants were then seated inside the Faraday cage, and a second saliva sample was obtained (Time 2 Cortisol). Participants then viewed the alarmist or control video (depending on group allocation). To maximize attention, participants were instructed to pay attention to the video as they would be required to answer questions about the video as part of a memory test at the conclusion of the study (though no memory test was conducted). After watching the video, participants again completed the STAI‐Y1 and RPQ, and a third saliva sample was obtained (Time 3 Cortisol). The exposure device was then set up, and the provocation trial commenced. The provocation trial began with a 5‐minute baseline interval, where the exposure device was not switched on and participants were asked to sit and relax and complete a symptoms and exposure status (SESS) questionnaire. This was followed by a 15‐minute exposure interval (RF‐OFF or RF‐ON, depending on group allocation), where participants were required to complete the SESS 12 minutes into the exposure interval. The provocation trial concluded with a 5‐minute post‐exposure interval, where participants provided a fourth saliva sample (Time 4 Cortisol) and completed the SESS 4 minutes into the post‐exposure interval. At each of the baseline, exposure, and post‐exposure intervals, a Nardalert S3 dosimeter was used to demonstrate to the participant the status of the exposure system. Upon completion of the provocation trial, participants completed the final STAI‐Y1 and RPQ, and were then guided out of the Faraday cage and asked if they had any concerns or questions about any aspects of the experiment. No participants reported any concern about the experiment.

### Data analyses

Data was analyzed using IBM SPSS for Windows Version 29 (IBM, Armonk, New York). Inspection of the data revealed that one participant did not complete the testing material correctly, that four participants reported incorrect belief about exposure (they believed they were being exposed (RF‐ON) when they were in the RF‐OFF condition (N = 1) or that they were not being exposed (RF‐OFF) when they were in the RF‐ON condition (N = 3), and thus for these participants the experimental manipulation did not work), that there was a technical issue with the exposure device (N = 1), and that two participants' saliva samples were contaminated with blood. As this data may have added noise and masked any potential effects from being detected, these participants (N = 8) were excluded from further analyses and were replaced to preserve counterbalancing. The final sample consisted of 144 participants (36 participants per group).

The data was inspected for normality via visual inspection and a series of Shapiro–Wilk tests. These inspections revealed that only the neuroticism score was normally distributed. To address this, the change in SESS symptom score data (SESS Exposure minus SESS Baseline) and the change in salivary cortisol response (Time 4 Cortisol minus Time 1 Cortisol) were normalized using a Two‐Step approach (Templeton, 2011). This approach involves transforming each variable into a percentile rank, which creates a uniform distribution. An inverse‐normal transformation was then applied to the percentile ranks to form a normal distribution.

A limitation of the two‐step approach to normalization is that it is only successful to the extent that the variable of interest is continuous in nature, and it is limited in its ability to transform continuous data that have a small number of levels, such as those measured on a five‐ or seven‐point Likert scales (Templeton, 2011). Therefore, where assumptions of normality were violated for the questionnaire data using Likert scales, including state and trait anxiety scores and risk perception scores, non‐parametric tests were employed. Where data points were missing for the STAI and NEO‐FFI, data were interpolated to preserve counterbalancing. Interpolated values were calculated by multiplying the total mean by the proportion of variance explained by gender (total mean divided by gender mean) and the proportion of variance explained by group allocation (total mean divided by group mean).

### Statistical analyses

Statistical analyses were conducted using IBM SPSS for Windows Version 29 (IBM, Armonk, New York). A series of preliminary analyses was first conducted to verify whether the four groups were comparable, and that the experimental manipulation was successful. For the hypothesis‐driven analyses, the dependent variables were the change in SESS symptom score (SESS Exposure minus SESS Baseline) and the change in salivary cortisol (Time 4 Cortisol minus Time 1 Cortisol). The independent variables were video (alarmist or control) and RF‐EMF exposure condition (RF‐ON or RF‐OFF).

#### Preliminary analyses

Kruskal‐Wallis tests were used to compare pre‐existing (T1) levels of state anxiety (STAI‐Y1), trait anxiety (STAI‐Y2), and risk perception (RPQ), separately, between each of the four experimental groups. A one‐way analysis of variance (ANOVA) was used to compare baseline salivary cortisol (T1 Cortisol) between each of the four experimental groups.

To verify whether the experimental manipulation of the video was successful, a Mann–Whitney U test was conducted to test whether the change in Risk Perception (RPQ T2 minus RPQ T1), was higher for participants who viewed the alarmist video (Group 3 and Group 4) compared to participants who viewed the control video (Group 1 and Group 2). Effect size was calculated as a *r* (Field, 2009).

To verify whether watching an alarmist media report increased symptoms, and separately, whether being openly exposed to RF‐EMF increased symptoms, a 2 × 2 Factorial ANOVA was used where SESS was the dependent variable, and the main effect of video condition (Alarmist; Control) and exposure condition (RF‐OFF; RF‐ON) was assessed. Effect size was calculated as omega‐squared (ω^2^) (Field, 2009).

#### Hypothesis driven analyses

To determine whether any effect on symptom score that being openly exposed to RF‐EMF had was related to the type of video watched, the interaction terms of the 2 × 2 Factorial ANOVA in the preliminary analyses were used, where SESS was the dependent variable, and the interaction of exposure condition (RF‐ON; RF‐OFF) and video type (Alarmist; Control) was assessed. Effect size was calculated as omega‐squared (ω^2^) (Field, 2009).

To determine whether viewing an alarmist video had any effect on cortisol response, whether being openly exposed to RF‐EMF had any effect on cortisol response, and whether any effect on cortisol response that being openly exposed to RF‐EMF had was related to the type of video watched, a 2 × 2 factorial ANOVA was used. Salivary cortisol was the dependent variable, and the main effects of video type (Alarmist; Control) and exposure condition (RF‐OFF; RF‐ON), and the interaction of exposure condition and video type were assessed. Effect size was calculated as omega‐squared (ω^2^) (Field, 2009).

A one‐tailed Pearson's correlation (*r*_*p*_ *)* was used to determine whether there was a positive relation between symptom change (SESS Exposure minus SESS Baseline) and salivary cortisol change (Time 4 Cortisol minus Time 1 Cortisol).

#### Exploratory analyses

A one‐tailed Spearman's Rho correlation (*r*_*s*_ *)* was used to determine whether there was a positive relation between baseline state anxiety and change in SESS symptom score (SESS Exposure minus SESS Baseline).

A one‐tailed Spearman's Rho correlation (*r*_*s*_ *)* was used to determine whether there was a positive relation between trait anxiety and change in SESS symptom score (SESS Exposure minus SESS Baseline).

A one‐tailed Pearson's correlation (*r*_*p*_ *)* was used to determine whether there was a positive relation between neuroticism (total of 12 corresponding neuroticism items on NEO‐FFI) and change in SESS symptom score (SESS Exposure minus SESS Baseline).

## RESULTS

### Preliminary analyses

The descriptive and test statistics for assessing whether there were significant differences between the four experimental groups in relation to pre‐existing levels of state anxiety, trait anxiety, risk perception, and baseline salivary cortisol are presented in Table 1. No significant differences were detected, indicating that there were no pre‐existing differences between the groups on these measures that may have confounded the results. Verifying that the video manipulation worked as intended, the change in RPQ score was higher for participants who watched the alarmist video (*Median* = 6) compared to participants who watched the control video (*Median* = 0), *U* = 4481.00, *z* = 7.638, *p* < .001, *r* = 0.64.

**TABLE 1:** Descriptive statistics and tests for differences in pre‐existing levels of state anxiety, trait anxiety, risk perception, and salivary cortisol between the four experimental groups.

Dependent variable	Group 1	Group 2	Group 3	Group 4	Test statistic
	N = 36	N = 36	N = 36	N = 36	
**State anxiety**	*M*_*r*_ = 67.96	*M*_*r*_ = 67.26	*M*_*r*_ = 66.29	*M*_*r*_ = 88.49	*H* (3) = 7.09, *p* = .069
**Trait anxiety**	*M*_*r*_ = 66.94	*M*_*r*_ = 65.53	*M*_*r*_ = 70.96	*M*_*r*_ = 86.57	*H* (3) = 5.80, *p* = .122
**Risk perception**	*M*_*r*_ = 74.47	*M*_*r*_ = 60.79	*M*_*r*_ = 73.40	*M*_*r*_ = 81.33	*H* (3) = 4.59, *p* = .205
**Salivary cortisol**	*M =* .20, *SD =* .17	*M =* .23, *SD =* .15	*M =* .30, *SD =* .19	*M = *.20, *SD =* .17	*F* (3, 140) = .98, *p* = .403

**Group 1 =** control video, RF‐OFF; **Group 2 =** Control Video, RF‐ON; **Group 3 =** Alarmist Video, RF‐OFF; **Group 4 =** Alarmist Video, RF‐ON; ***M***_***r***_ **= **mean rank; ***M =*** mean; ***SD* =** standard deviation.

There was no difference in the change in SESS symptom score between participants who viewed the alarmist video (*M* = 53.63, *SD* = 186.66) compared to participants who viewed the control video (*M* = 36.51, *SD* = 190.10), *F* (1, 140) = .39, *p* = .53, ω^2^ = −.003. Participants in the RF‐ON condition had a greater increase in symptoms (*M* = 138.05, *SD* = 191.70) than participants in the RF‐OFF condition (*M* = −47.90, *SD* = 129.79), *F* (1, 140) = 45.95, *p* < .001, ω^2^ = .24.

### Hypothesis driven analyses

#### The effect of video and exposure conditions on the change in symptom score

**FIGURE 3:**
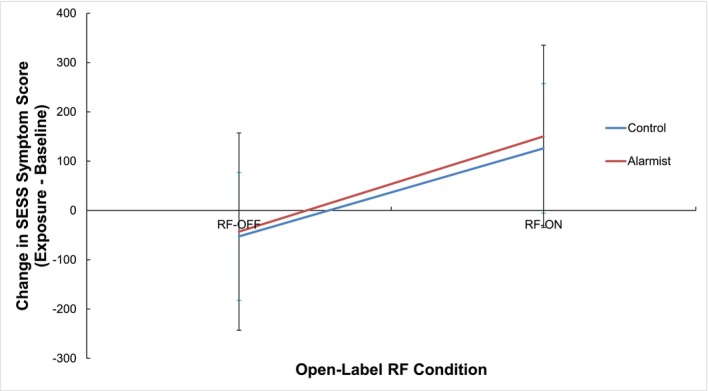
The change in SESS symptom score (exposure – baseline) as a function of video group and exposure condition. Error bars represent standard deviation.

Figure 3 shows the change in SESS symptom score as a function of video condition and open‐label exposure condition. The difference in SESS symptom score between the RF‐ON and RF‐OFF conditions was not affected by whether participants viewed the alarmist or the control video, *F* (1, 140) = .07, *p* = .79, ω^2^ = −.005.

#### The effect of video and exposure conditions on the change in cortisol

**FIGURE 4:**
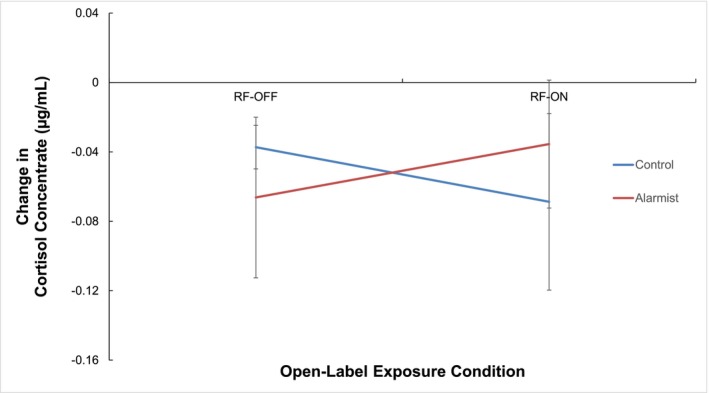
The change in salivary cortisol response (exposure – baseline) as a function of video group and exposure condition. Error bars represent standard deviation.

Figure 4 shows the change in salivary cortisol as a function of video condition and open‐label exposure condition (in μg/dL). There was no significant difference in the change in salivary cortisol between participants who viewed the alarmist video (*M* = −.05, *SD* = .16) compared to participants who viewed the control video (*M* = −.05, *SD* = .13), *F* (1, 140) = .02, *p* = .894, ω^2^ = −.006. There was no significant difference in the change in salivary cortisol between participants in the RF‐ON condition (*M* = −.05, *SD* = .13) compared to participants in the RF‐OFF condition (*M* = −.04, *SD* = .16), *F* (1,140) = .27, *p* = .605, ω^2^ = −.005. The difference in salivary cortisol between the RF‐ON and RF‐OFF conditions was not affected by whether participants viewed the alarmist or the control video, *F* (1, 140) = 2.15, *p* = .145, ω^2^ = .008.

#### The association between change in symptom score and change in cortisol response

A significant correlation between change in symptom score and change in salivary cortisol was not identified, *r*_*p*_ = .04, *p* = .316.

### Exploratory analyses

#### The association between baseline state anxiety and change in symptom score

A significant correlation between pre‐existing state anxiety and change in symptom score was not identified, *r*_*s*_ = .074, *p* = .190.

#### The association between trait anxiety and change in symptom score

A significant correlation between trait anxiety and change in symptom score was not identified, *r*_*s*_ = .110, *p* = .094.

#### The association between neuroticism and change in symptom score

A significant correlation between neuroticism and change in symptom score was not identified, *r*_*p*_ = .136, *p* = .052.

## DISCUSSION

Although there has been consistent evidence that the symptoms reported by IEI‐EMF sufferers are likely the result of a nocebo effect, and that explicit suggestions of harm from alarmist media may contribute to this nocebo effect, criticisms about the subjective nature of the questionnaires used in these studies have been raised by some scientists and many IEI‐EMF advocacy groups (Bräscher et al., 2020; Leszczynski, 2021). Therefore, the present study aimed to, first, replicate the results of our previous study which showed that viewing an alarmist video and being openly exposed to RF‐EMF increased symptoms in healthy participants, and second, extended this to determine whether being openly exposed to RF‐EMF after viewing an alarmist video can induce an associated cortisol response in healthy participants.

Preliminary tests first verified that there were no differences in pre‐existing levels of state anxiety, trait anxiety, risk perception, neuroticism or baseline salivary cortisol between the four experimental groups, that the alarmist video manipulation worked as intended by increasing participant's risk perception, and that participants who were aware that they were being exposed had an increase in symptoms compared to participants who were aware they were not being exposed. These verifications were not only important for establishing that any subsequent differences between groups could be confidently attributed to the experimental manipulations, but also to confirm that being openly exposed to RF‐EMF increased symptoms consistent with effects found in the broader IEI‐EMF literature (Eltiti et al., 2007; van Moorselaar et al., 2017; Verrender, Loughran, Anderson, et al., 2018; Verrender, Loughran, Dalecki, et al., 2018).

Interestingly, the current study failed to find an effect of viewing an alarmist media report and being openly exposed to RF‐EMF on symptoms. This was unexpected and contrasts with previous studies, which have shown that viewing alarmist media reports contributes to experiencing a nocebo effect (Bräscher et al., 2017; Verrender, Loughran, Dalecki, et al., 2018; Witthöft & Rubin, 2013). Recently, however, Bräscher et al. (2020) reported that a nocebo effect, where participants reported an increased self‐reported intensity and aversiveness to an electrodermal stimulus during a Sham Wi‐Fi signal, was not dependent on viewing an alarmist media report. Like Bräscher et al. (2020), it is possible that contextual factors in the current study, such as the information given during the study briefing and the study environment (Faraday cage, RF‐EMF antennas, RF‐EMF dosimeter, being told that they were being exposed), may have been more important for eliciting a nocebo effect than the effect of viewing the alarmist media report.

The current study also failed to find an effect of viewing an alarmist media report and being openly exposed to RF‐EMF on salivary cortisol, and no relationship between salivary cortisol and symptoms was detected. This suggests that the HPA axis may not be affected by viewing an alarmist media report or experiencing an EMF exposure situation that could be perceived as threatening. However, it is possible that time on task effects, or diurnal variation in cortisol secretion, may have influenced the results by increasing error variance and thus reducing statistical power. It is also possible that the anticipation of participating in an RF‐EMF provocation study may have elevated salivary cortisol prior to testing, resulting in a possible ceiling effect. Further testing would be required to clarify whether these limitations importantly affect the results. In addition, despite being powered to detect a medium sized effect on salivary cortisol (based on the results of a study which tested the effect of a high valence film on salivary cortisol response [Nejtek, 2002]), the current study may not have had sufficient statistical power to detect small effects from a one‐time alarmist media communication about RF‐EMF. Indeed, in the present study, the observed effect sizes for the salivary cortisol outcomes were below ω^2^ = .008, which is so small that it would not be feasible to conduct a study to determine whether these effects are significant at the *p* < .05 level (e.g., it would require a sample size of at least N = 971).

The ability to identify individual attributes that may contribute to people being more susceptible to nocebo effects has important clinical ramifications, as it may help with the development of effective, tailored interventions. In the current study, we explored whether pre‐existing state anxiety, trait anxiety, or neuroticism were related to increased symptom reporting. Although no relationship between pre‐existing state anxiety and symptoms was found, it is difficult to conclude whether there may be a potential relationship between trait anxiety and symptoms, and neuroticism and symptoms, given the trend level relationships detected. While it is clear that further testing is required to elucidate whether neuroticism and trait anxiety are related to experiencing a nocebo response, our results correspond with the broader nocebo literature, which suggests that it is difficult to reduce potential contributors to the nocebo response to a single factor, especially when investigating nocebo responses in healthy, predominantly young participants (Kern et al., 2020; Webster et al., 2016). It is still possible that patient populations, including those who experience IEI‐EMF, present with personality characteristics which may interact with other dispositional and contextual factors, which lead to the experience of a nocebo response, though this is speculative and needs to be investigated further.

Several important issues also remain to be clarified. First, as the current study utilized a single, open‐label provocation trial, it is difficult to differentiate any possible effect that exposure to RF‐EMF may have had on cortisol response, independent of a nocebo effect or a participant's belief that they were being exposed. To determine whether exposure to RF‐EMF independently affects cortisol response, and to clarify the role of knowledge of exposure status in influencing cortisol response, future studies would need to incorporate a double‐blind protocol with both a sham and active exposure condition. Second, it is possible that the anticipation of participating in a provocation study may have elevated salivary cortisol, leading to a ceiling effect. This may have masked any potential effect of the alarmist media report and open‐label exposure condition on salivary cortisol response being detected. To clarify whether this is an important methodological consideration, it would be useful for future studies to include a pre‐trial‐day baseline saliva sample and test whether salivary cortisol is higher on the provocation trial day compared to the pre‐trial day.

This was the first study to test whether viewing an alarmist media report and being openly exposed to RF‐EMF could induce a salivary cortisol response and to test whether this stress response was associated with an increase in symptoms in healthy participants. Consistent with previous studies, there was clear evidence that the knowledge or awareness of being exposed to RF‐EMF increased symptoms in healthy participants. However, the current study failed to find an effect of viewing an alarmist media report and being openly exposed to RF‐EMF on symptoms and salivary cortisol, and no relationship between salivary cortisol and symptoms was detected. In conclusion, this and previous research, suggests that the nocebo effect associated with the symptoms attributed to RF‐EMF exposure, in healthy controls, may be more closely associated with higher‐level than lower‐level neural processing, where awareness and belief of exposure seem to play a more important role in symptom perception than underlying physiological processes (Van den Bergh et al., 2017).

## CONFLICT OF INTEREST STATEMENT

Sarah P. Loughran receives funding from the National Health and Medical Research Council of Australia (NHMRC). She is the Director of Radiation Research and Advice at the Australian Radiation Protection and Nuclear Safety Agency (ARPANSA), a member of the Scientific Expert Group at the International Commission on Non‐Ionizing Radiation Protection (ICNIRP), and a member of the World Health Organisation Task Group on Radiofrequency Fields and Health Risks. Rodney J. Croft receives funding from the National Health and Medical Research Council of Australia (NHMRC) and is a member of the Scientific Expert Group at the International Commission on Non‐Ionizing Radiation Protection (ICNIRP). No potential competing interests were reported by the remaining authors.

## ETHICS STATEMENT

This study was approved by the University of Wollongong Human Research Ethics Committee (HE: 2022/146).

## Data Availability

The data that support the findings of this study are available via PsycArchives (DOI: 10.23668/psycharchives.16320).
